# Effects of Nanosilica on the Properties of Ultrafine Cement–Fly Ash Composite Cement Materials

**DOI:** 10.3390/nano14241997

**Published:** 2024-12-13

**Authors:** Kai Wang, Siyang Guo, Jiahui Ren, Pengyu Chen, Qihao Zhang

**Affiliations:** School of Civil Engineering and Architecture, Henan University, Kaifeng 475000, China; siyangguo98@163.com (S.G.); renjiahui159@henu.edu.cn (J.R.); 19503903296@163.com (P.C.); 18272561299@163.com (Q.Z.)

**Keywords:** ultrafine cement, ultrafine fly ash, nanosilica, grouting material, orthogonal tests

## Abstract

The increasing incidence of structural failures, such as cracks and collapses, in rock masses within mines, tunnels, and other civil engineering environments has attracted considerable attention among scholars in recent years. Grouting serves as a principal solution to these issues. The Renlou Coal Mine in the Anhui Province is used as a case study to evaluate the effectiveness of nanosilica (NS) as an additive in ultrafine cement (UC), introducing a novel grouting material for practical applications. This study investigates the physical and microscopic properties of a UC–ultrafine fly ash (UFA) mixed slurry containing powdered NS. Slurries of pure UC, UFA-blended UC, and UFA-blended UC with NS were prepared, and their viscosity, water precipitation rate, and compressive strength were evaluated. Scanning electron microscopy and X-ray diffraction were used for microscopic analyses. The results showed that the addition of UFA and NS to the UC slurry induced a more compact structure with reduced porosity. It was found that the viscosity and 7 d and 28 d compressive strengths of the slurry containing 50% UFA decreased by 91%, 51%, and 29.2%, respectively, and the water separation rate increased by 306.5%. The decrease in early strength was more pronounced, and the UFA content should not exceed 25%. Compared with the slurry without NS, the viscosity and 7 d and 28 d compressive strength of the slurry containing 1.5% NS increased by 216%, 51.2%, and 37%, respectively, and the water separation rate decreased by 45%. Notably, when the NS content is 1.5%, the performance of cement slurry is improved the most, and more C-S-H gel is produced. Cement consumption costs could be lowered and slurry performance improved by replacing a part of the cement with UFA and NS. Finally, orthogonal tests were conducted to select the optimal proportions for cement grouting. The optimal blend was determined to be composed of 20% UFA and 1.5% NS, with a water–cement ratio of 0.6. The study’s results not only demonstrate that NS has a good effect on improving the performance of cement-based grouting materials but also provide new insights for the design and application of grouting support in underground engineering.

## 1. Introduction

**Figure 1:**
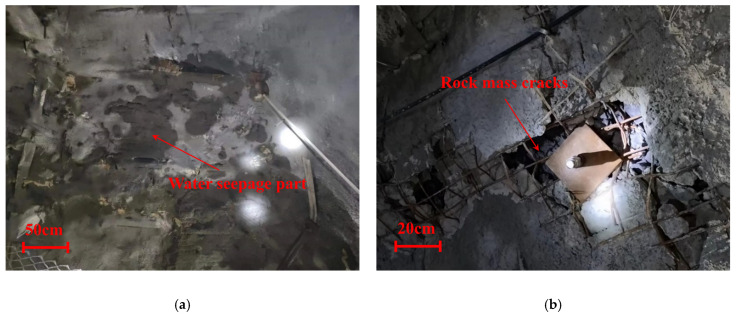
“7_3_53” project overview. (**a**) Roof seepage and (**b**) rock mass cracks.

As the national economy undergoes rapid expansion, there is a concurrent acceleration in the construction of underground projects, such as subways, tunnels, and mines. Consequently, safety standards are escalating to meet the challenges posed by adverse construction environments. Geotechnical engineering reinforcement plays a crucial role in its stability and safety, especially during the construction process. Many microcracks and holes appear in the rock body and gradually form macrodamaged structures, which reduce the strength of the rock and impact engineering safety [1]. Following the increasing depletion of shallow underground resources, underground engineering has begun to move deeper. According to statistics, the mining depth of coal mines in China has exceeded 800 m, and some have even reached 1500 m [2]. Underground deep engineering often faces more difficulties, such as (among others) high temperature, high pressure, high moisture content, and high and low stress [3]. The number “7_3_53” working face of Renlou Coal Mine in Huaibei City, Anhui Province, China, was the subject of a case study. The “7_3_53” roadway refers to the centralized transportation roadway with the number “53” for excavating the “7_3_” coal seam. Onsite investigations revealed numerous cracks and water seepage in the surrounding rock of certain tunnels, as illustrated in Figure 1. These situations will likely lead to major accidents, causing casualties and economic losses. Grouting technology emerges as a viable solution to address this engineering challenge. Grouting equipment can inject slurry into the fractured rock body by using techniques such as extrusion, filling, and infiltration. The slurry can flow to a designated location where rapid condensation occurs, and the fissure rock body can then form an integral whole to enhance the strength of the rock body. Simultaneously, grouting can improve the densification and resistance to seepage so that the comprehensive performance of the rock body is enhanced [4]. In most cases, the main material used for grouting is Portland cement because it is easy to obtain, low-cost, highly functional, and has strong bonding strength [5]. However, during the grouting process, ordinary Portland cement may be subjected to high pressure for a long time, which may have adverse effects on the rheological properties of the slurry, and even cause bleeding, seriously affecting the construction quality [6]. In addition, Portland cement itself also has some internal effects, such as high hydration heat, large drying shrinkage, and incomplete early hydration [7]. These properties need to be strengthened to obtain more durable and better comprehensive performance cement grouting materials. Therefore, it is necessary to find a new type of cement-based grouting material to solve the aforementioned problems.

Given the growing complexity of engineering environments, the demand for grouting materials has increased. Cement is extensively used as a first choice for grouting in different engineering applications. However, conventional cement has limitations primarily stemming from its relatively large particle size. As a result, it can only effectively penetrate cracks and pores ≥ 0.1 mm, leading to suboptimal grouting performance, particularly for finer cracks [8]. Enhancing the groutability of cement-based grouts in low-permeability media, ultrafine cement (UC) grouting materials have become a popular research topic worldwide. UC exhibits comparable permeability and groutability, along with superior durability and strength compared with the corresponding properties of organic chemical grout, and it is also environmentally friendly and nonpolluting to the surrounding environment [9]. UC has demonstrated successful application in many projects, yielding favorable grouting results. The strength of cement is usually related to factors such as admixture, water–cement ratio (W//C), cement grade, preparation method, curing time, etc. [10,11]. Therefore, to improve the performance of cement, admixtures, such as fly ash, slag, and silica fume, are often added to it to achieve higher cement durability and strength. The cement’s strength can be improved by adding an appropriate amount of nanosilica (NS) to the cement as an admixture [12]. Therefore, this article uses NS as an additive to improve the performance of grouting materials. Many previous studies have confirmed that adding NS to cement can help improve the overall performance of the slurry [13,14].

Recent studies have been dedicated to the investigation of nanomaterials, with a particular focus on the enhancement of cementitious nanomaterials [15]. Nanomaterials are characterized by having at least one nanoscale dimension within the three-dimensional space, typically ranging from 1 to 100 nm [16]. Nanomaterials have small particle sizes, low densities, and superior physical and chemical properties. Incorporating an appropriate number of nanomaterials into cement can enhance its workability and durability, as well as improve its chemical reaction activity. NS is presently the most extensively used nanomaterial in cement [17]. NS can enhance numerous properties of cementitious materials, such as cohesion and resistance to cracking due to internal water loss, mechanical strength, and durability [18]. NS not only serves as a filler for cementitious materials but also promotes the volcanic ash reaction and can provide nucleation sites for hydration products during the early stages of hydration reactions, improving the compactness of the material. NS possesses a higher specific surface area compared with silica fume, and it exhibits an effective volcanic ash reaction [19]. Therefore, NS can facilitate the early hydration of cement and result in higher early strength. Achieving good dispersion of NS is key to enhancing the properties of cementitious materials, and the mechanical properties of cementitious materials are influenced by the dispersion of NS particles within them [20]. When NS is used in cement, it induces the agglomeration of NS particles, which absorb a considerable amount of free water. This insufficient hydration of the cement leads to an adverse effect on its performance [21,22], because NS replaces some cement and reduces the CO_2_ generated by cement hydration reactions. Therefore, adding NS can also reduce CO_2_ emissions and does not compromise the durability of cementitious materials, especially when combined with fly ash (FA) [23]. However, studies have demonstrated that incorporating blends of NS and FA into cement yields early and late strengths comparable to those of pure cement, while simultaneously reducing the amount of cement used and lowering CO_2_ emissions compared with pure cement [24,25]. Korniejenko et al. [26] concluded that NS is one of the most popular and the most promising cement additives to achieve results.

Du et al. [27] found that for ordinary and ultralightweight cement, when the NS content exceeds 2%, it will cause the NS to agglomerate, increasing the gas content in the cement and a decrease in strength. Zhang et al. [28] found that adding NS to cement can greatly improve the mechanical properties of the slurry, with a 20.6% increase in bonding ability. However, NS has an adverse effect on shrinkage performance, which can be offset by adding a large amount of ultrafine fly ash (UFA). Yang et al. [29] found that uniformly dispersed NS is more conducive to improving the mechanical properties of cement-based materials. When the NS content is 0.4%, the compressive strength at 3 and 28 d increased by 5.86% and 8.94%, respectively. Ye [30] investigated the impact of NS content on the mechanical properties of cement-based materials and observed that a low content of NS can enhance considerably the flexural and compressive strengths of such materials, and showed that the mechanical properties peaked when the mass fraction was 3%. Kim et al. [31] pointed out that adding NS and silica fume to cement with a high FA content accelerated the early hydration reaction of the slurry, thereby shortening the setting time and improving the compressive strength in the early and middle stages of hydration. By contrast, studies have also found that the use of NS reduces the bleeding of concrete, prolongs the setting time, and enhances the cohesiveness of freshly mixed concrete [32]. NS can react with Ca(OH)_2_ (CH) in pozzolanic reactions at an early stage. Owing to the pozzolanic activity of CH, CH can be transformed into C-S-H gel, improving the mechanical properties of concrete [33,34]. Based on X-ray diffraction (XRD) and scanning electron microscopy (SEM) experiments, it was found that the amount of CH decreased with the increase in the NS content. The C-S-H and CH produced by cement slurry containing 10% NS increased by 66% and 61%, respectively, within 8 h. The C-S-H and CH produced during the 24 h hydration reaction decreased by 25% and 32%, respectively [35]. Rostami et al. [36] found that the dispersion degree of NS in cement slurry can affect the fresh mixing performance of the slurry. Usually, it is crucial to determine whether NS has good dispersibility, as well-dispersed NS can optimize the performance of cement-based materials [37]. Abhilash et al. [38] found that an NS dosage of ≥3% may lead to an increase in porosity, microcracks, and lower compressive strength of the slurry, which is caused by the agglomeration of NS particles. Du et al. [39] found that more than 1% NS increased the viscosity porosity of cement slurry, as air would enter the interior parts of the slurry during the mixing process, causing more air to enter the mixture. If the filling effect of NS particles is not sufficient to compensate for the increased air content inside the slurry, it will lead to an increase in porosity inside the hardened slurry. 

There are many studies on the application of NS in cementitious materials, but there is lacking research on the improvement of grouting material properties by adding NS to cement-based materials. In previous studies, the optimal amounts of added NS in cement-based materials had been inconsistent, which may affect its application in the field of grouting [40,41,42]. In large-scale engineering, problems such as cracks, collapses, and water seepage in rock masses and walls cannot be ignored. Therefore, it is particularly important to identify a new grouting material with high performance. The purpose of this study is to investigate the influence of NS on the physical and microscopic properties of cement slurry at a certain dosage and to conduct viscosity, water separation rate, and strength tests to determine the fresh mixing characteristics and hardening degree of cement slurry to apply grouting technology more effectively in practical engineering applications. Therefore, this study determines the optimal ratio of various materials in cement-based materials through orthogonal experiments, and conducts grouting experiments in practical engineering, achieving good grouting effects. This has important implications for the field of grouting. Accordingly, this study proposes a novel approach for developing grouting materials, offering strategic insights and designs to enhance the performance of cement grouting materials in practical engineering applications, and guiding future research in the field of cement grouting.

## 2. Materials and Methods

### 2.1. Materials

**Figure 2:**
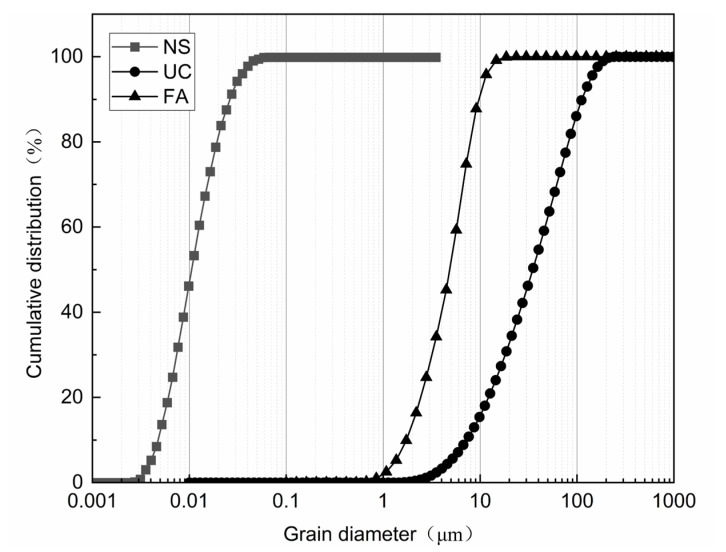
Particle size distributions of materials.

UC was procured from Kangjing New Material Technology Co., Ltd., Shandong, China, UFA was sourced from Henan Yulian Energy Group Co., Ltd., Gongyi, China, NS was obtained from Yaoyi Alloy Materials Co. Ltd., Shanghai, China, and their composition is listed in Table 1. The chemical compositions of UC, UFA, and NS were provided by the original manufacturers. A high-performance polycarboxylic acid water-reducing agent was also selected. The particle size distributions of the UC, UFA, and NS were measured using a Mastersizer 3000 laser diffraction (Shanghai Sibeiji Instrument Co., Ltd., Shanghai, China) particle size analyzer, as depicted in Figure 2. The results show that the proportion of UC with a particle size < 100 μm accounted for 86%. Additionally, the proportion of UFA with a particle size < 10 μm accounted for 87.77%, whereas that of NS particles with a diameter < 0.01 μm accounted for 46.16%.

### 2.2. Experimental Method

This study designed six sets of experiments, as summarized in Table 2. Viscosity, water separation rate, and strength tests were performed on each group of experiments. Three samples were prepared for each test and the average was estimated to obtain the final test results. The test results are listed in Table 3. In this experiment, the NS dosage was controlled within 1.5%. To maintain consistency in the experiment, a 1% water-reducing agent was added to each group of experiments, and the water–cement ratio was controlled at 0.6 for each group of experiments. In addition, to ensure good dispersibility of the NS solution, it is necessary to use an ultrasonic cleaning machine to disperse the NS solution ultrasonically.

**Figure 3:**
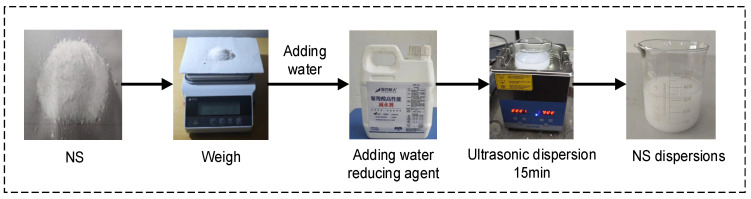
NS dispersion production process.

Ultrasonic dispersion of NS solution: We used the SN-QX-20D ultrasonic cleaning machine produced by Shanghai Shangpu Instrument Equipment Co., Ltd. in Shanghai China to ultrasonically disperse the NS solution. The dispersion method involved the sequential addition of appropriate amounts of NS and water to a beaker, stirring of the solution evenly with a glass rod, the addition of 1% water reducer, stirring evenly with a glass rod again, and the final placement of the beaker containing the NS solution into an ultrasonic cleaning machine for ultrasonic dispersion. The ultrasonic frequency was 40 ± 2 kHz, the temperature was 22 °C, and the dispersion time was 15 min. The production process of NS dispersion is shown in Figure 3.

**Figure 4:**
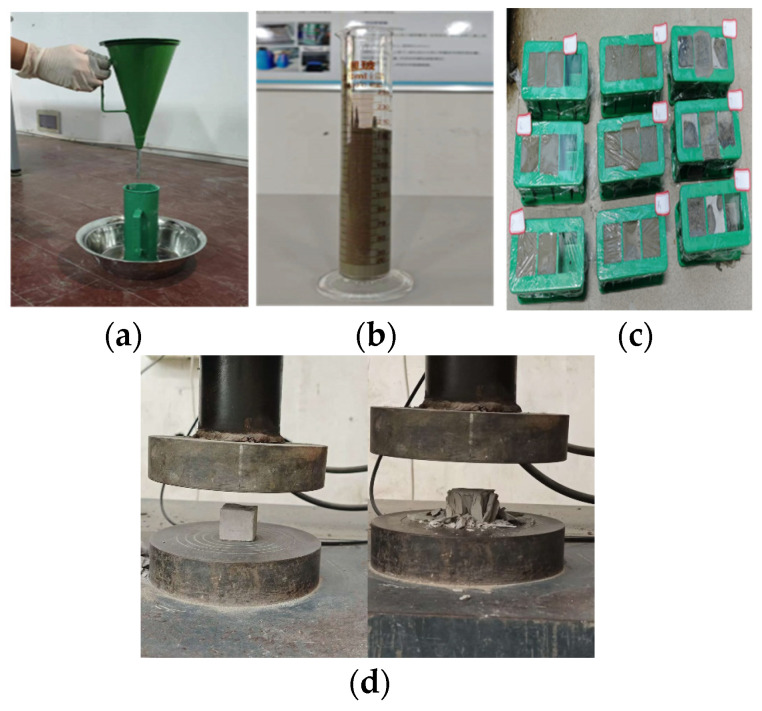
Physical performance tests of cement materials. (**a**) Viscosity, (**b**) water separation rate, (**c**) test piece maintenance, and (**d**) compressive strength tests.

Viscosity test: Using a martensitic funnel viscometer as in Figure 4a, tests were conducted in accordance with the specification of the American Society for Testing and Materials standard D6910/D6910M-09 [43]. The testing method involved the pouring of 700 mL of slurry into a funnel, the placement of a 500 mL measuring cup under the funnel, and the initiation of time recordings with a stopwatch at the instant the slurry started to flow out from the funnel. The timing ended when the slurry filled the 500 mL measuring cup. The recorded time is associated with the viscosity of the measured slurry. Each set of slurry was tested three times, and the final result is the average of these tests. Water precipitation test: The prepared slurry was poured into a 200 mL measuring cylinder, as shown in Figure 4b. The cement particles were allowed to settle for 2 h, and the volume of water was recorded when the upper clear water level no longer increased. Each batch of slurry underwent three separate water separation rate tests, with the final result being the average of these measurements. Condensation time test: A Vicat instrument was used to determine the initial and final setting times according to the specifications of the GBT1346-2019 standard [44]. Experimental sample preparation: The cement slurry was prepared according to the ratio given in Table 2. After mixing the slurry evenly with a cement slurry mixer, the mixed slurry was poured into a three-connected mold with a standard size of 40 mm × 40 mm × 40 mm, as shown in Figure 4c. It was then sealed with cling film, the cling film was removed after 1 d, and it was cured under standard curing conditions (temperature 20 ± 2 °C; relative humidity > 95%) for 7, 14, and 28 d, respectively. In total, 54 samples were prepared for the subsequent experiments. Compressive strength test: The test was conducted in accordance with the standard GBT17671-2021 [45]. The compressive strength was tested at 7, 14, and 28 d, respectively, as shown in Figure 4d. When testing compressive strength, each group of cement specimens underwent three tests, and the compressive strength values were averaged.

**Figure 5:**
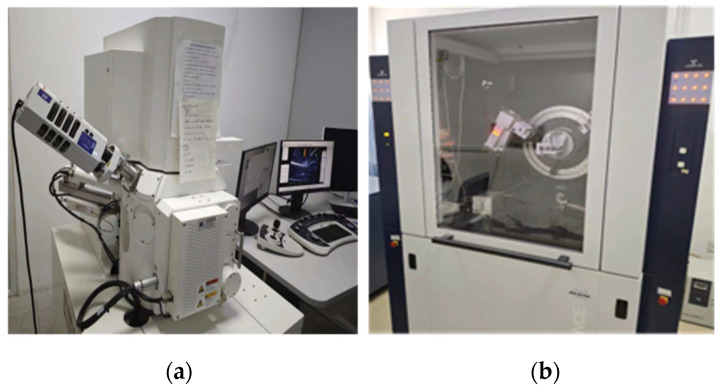
Cement material microperformance testing devices: (**a**) scanning electron microscopy and (**b**) X-ray diffraction analysis.

**Figure 6:**
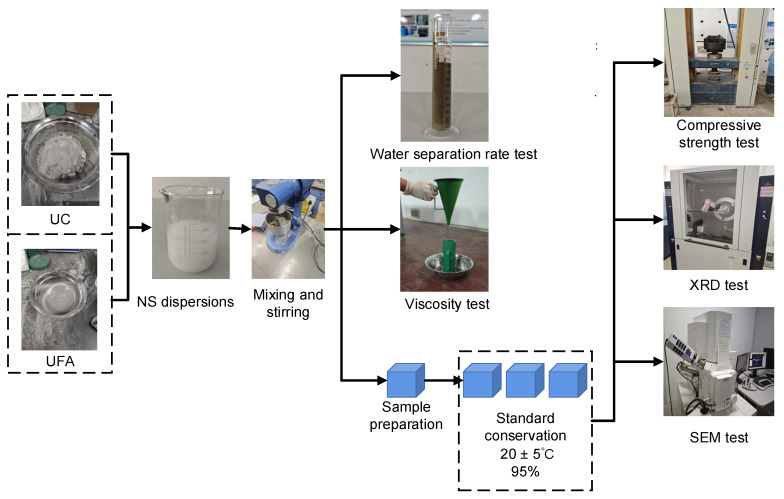
Outline of the entire experimental procedure.

As shown in Figure 5a, we used field-emission scanning electron microscopy tests on samples with a curing age of 28 days to observe and analyze the microscopic morphology of each group of samples. Before the SEM test, the samples were prepared into small flakes measuring 5 mm by 5 mm, and the samples were immersed in anhydrous ethanol for 24 h to halt hydration, and their surfaces were gold-sprayed. An XRD line powder diffractometer (D8 Advance) was used to determine the mineral compositions after 7 and 28 d, as shown in Figure 5b. Before the XRD test, the samples were soaked in anhydrous ethanol for 24 h to halt hydration; after the test, they were ground into powder for further testing. Figure 6 outlines the overall test procedure.

Finally, an orthogonal test was performed to select the slurry proportion with optimal comprehensive performance. In this experiment, three factors and three levels were selected: UFA dosages of 20%, 30%, and 40%; NS dosages of 0.5%, 1%, and 1.5%, and W/Cs of 0.6, 0.7, and 0.8. The orthogonal experimental design plan “L9 (3^3^)” was used, as indicated in Table 4.

## 3. Results and Discussion

### 3.1. Viscosity

**Figure 7:**
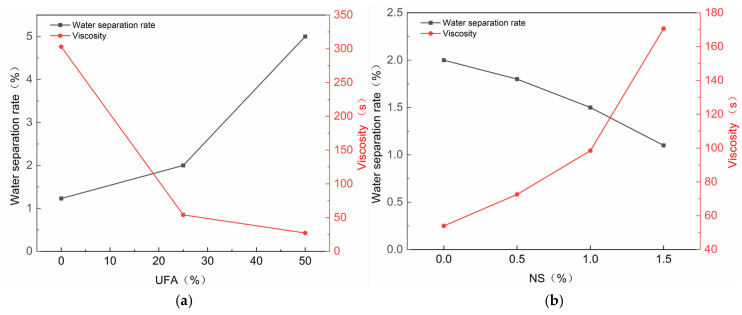
Effects of UFA and NS dosing on slurry performances: (**a**) UFA; (**b**) NS mixed with 25% UFA.

**Figure 8:**
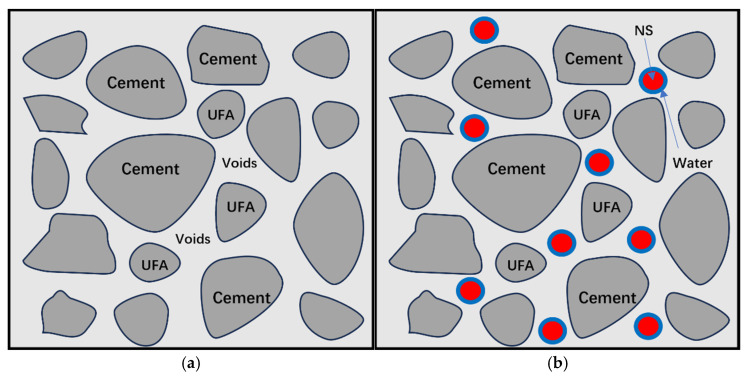
The mechanism of action of UFA and NS: (**a**) cement–fly ash, and (**b**) cement–fly ash–silica fume.

The viscosity of the slurry is a critical determinant of its fluidity. The test results are presented in Table 3. As shown in Figure 7a, as the UFA content increases up to 25%, there is a notable reduction in viscosity, thereby enhancing the slurry’s fluidity. Figure 8a shows the mechanism of action of UFA in cement. Based on these results, it can be observed that UFA can fill the gaps between cement particles, playing a filling role and increasing the density of the slurry. This is consistent with the findings of previous studies, where the porosity in cement containing 5% to 20% fly ash (FA) was reduced by 17% to 23%, and the cement’s void ratio decreased with an increasing percentage of FA [46]. Fly ash reduces the friction between cement particles and increases the fluidity of the slurry [47]. 

As shown in Figure 7b, as the NS content increases, the viscosity of the slurry also increases, leading to decreased fluidity. The viscosity tests suggest that the increase in viscosity remains relatively stable when the NS content does not exceed 1%. This is similar to previous research results, where the viscosity of cement grouting materials increased as a function of the NS content when the water–cement ratio was between 0.5 and 0.75 [48]. Because NS can fill the gaps that UFA cannot fill, it can further improve the compactness of the slurry. However, owing to their large specific surface area and high surface energy, NS are prone to agglomeration. The NS moieties that gather together cannot be filled into cement particles as fillers, and wrap around free water, thereby reducing the fluidity of the slurry [49]. Previous studies have found that as the content of NS increases, the cement paste forms lumps and absorbs a significant amount of water molecules, which does not provide a filling effect, leading to a decrease in the fluidity of cement-based materials [50]. In an ideal state, high-performance grouting materials should have lower viscosity and higher strength. Low-viscosity grouting materials can handle some small cracks in engineering [51]. Although ultrasonic dispersion was performed on the NS solution in this experiment, a large amount of NS still exists in the form of aggregation, and the addition of UFA can counteract this adverse effect.

### 3.2. Water Precipitation Rate

The precipitation rate is the key factor in determining the stability of the slurry; a lower precipitation rate means a more stable slurry. As shown in Figure 7a, when the UFA content increases, the water precipitation rate increases. When the UFA content exceeds 25%, the water separation rate increases considerably, which compromises the stability of the slurry. The increase in the water precipitation rate occurs because when UFA is added UFA, the UFA particles displace some cement particles and evenly fill the gaps, thus reducing the amount of filling water in the cement. Moreover, the UFA undergoes hydration reactions with water in the early stage, which increases the amount of free water and the water precipitation rate. Conversely, previous research has found that when the FA content is 8%, the viscosity coefficient of the mortar is 0.768 Pa·s, which is higher than that of the control group without FA [52]. This indicates that a low percentage of FA can improve the water separation rate of cement to some extent.

With the addition of NS, the precipitation rate gradually decreased, and the trend of decreasing water separation rate was relatively stable within a dosage of 1.5%. This is consistent with the previous research results [53]. When the NS content is between 1.5% and 3%, the water separation rate decreases from 4.5% to 1%, showing a downward trend. However, when the NS content is between 0.5% and 1%, the water separation rate slightly increases, which is different from the findings of this experiment. This decrease occurs because the specific surface area of the NS is large, there are many unsaturated bonds, and the NS particle size is small. Some free water combines with the surface of the NS crystals to form silicon hydroxyl (Si-OH) groups [54]. As shown in Figure 8b, NS can also effectively fill pores that UFA cannot fill, making the slurry denser and reducing its porosity, thereby decreasing the water precipitation rate of the slurry [55].

### 3.3. Compressive Strength

**Figure 9:**
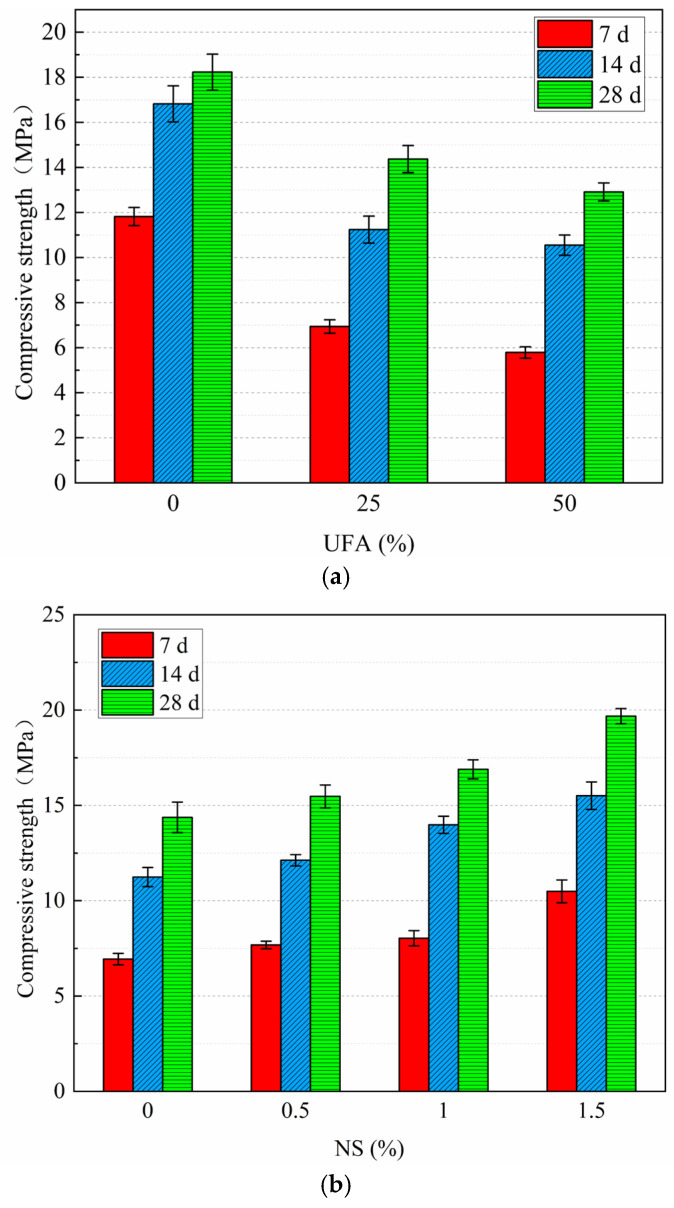
Effects of UFA and NS dosing on the compressive strength: (**a**) UFA and (**b**) NS mixed with 25% UFA.

The compressive strengths of the UFA and NS at different dosages are shown in Figure 9. Figure 9a shows that the strengths of the 7 and 14 d slurries decrease as the UFA content increases. However, the strengths of the 50% UFA slurry showed a greater reduction compared with the 25% UFA slurry. This is because the curing period of 7 d belongs to the early stage of cement strength development, whereas FA hardly participates in the reaction and does not produce strength. Moreover, a large amount of FA replaces cement and reduces the specific gravity of cement in the slurry; this leads to a reduction in the hydration products of cement in the early stage, thereby resulting in a reduction in strength in the first and middle stages [56]. Previous studies have similarly found that the addition of fly ash alone can reduce the compressive strength of cement, with a positive correlation between the amount of FA added and the decrease in strength. This is primarily due to the lower pozzolanic activity of FA [57]. When the curing age is 28 d, it is already at the later stage of the hydration reaction due to the fact that the volcanic ash reaction between FA and cement mainly occurs at the late stage when more hydration products are produced, which makes the pore structure denser and improves the strength at the later stage [58].

Figure 9b shows that the strength of the modified cement slurry increases at all stages after the incorporation of NS. In particular, when the NS dosage is 1.5%, the strength improvement is higher. Similarly, previous studies have shown that when 0.5% ≤ NS content ≤ 2%, the strength of all other cement with NS content is improved considerably [59]. However, if the NS content exceeds 3%, it may have adverse effects on the development of cement strength, and the optimal content of NS has also been determined to be 3% [60]. NS contributes to the development of early cement strength. This is because NS accelerates the hydration reaction and reacts with CH (in the cement) to produce C-S-H gel, which is an important component of strength. NS possesses high surface activity, which enhances the rate of hydration reactions, thereby allowing the products of hydration to accumulate rapidly in the pores. Consequently, the appropriate NS amount can improve the densification of modified cement slurry and reduce its porosity [59]. When the particle size is small, the nucleation effect of NS becomes more evident, and the number of nucleation sites increases, which contributes to the formation of C-S-H gels and improves their strength [61]. Adding UFA in the NS-doped slurry may also enhance the later strength of the slurry while reducing its viscosity. The active silica or aluminum silicate in the UFA reacts with the cement particles (according to a pozzolanic reaction) in the presence of water, thus forming C-S-H gel, which contributes to the development of strength [62].

### 3.4. Scanning Electron Microscopy Test

**Figure 10:**
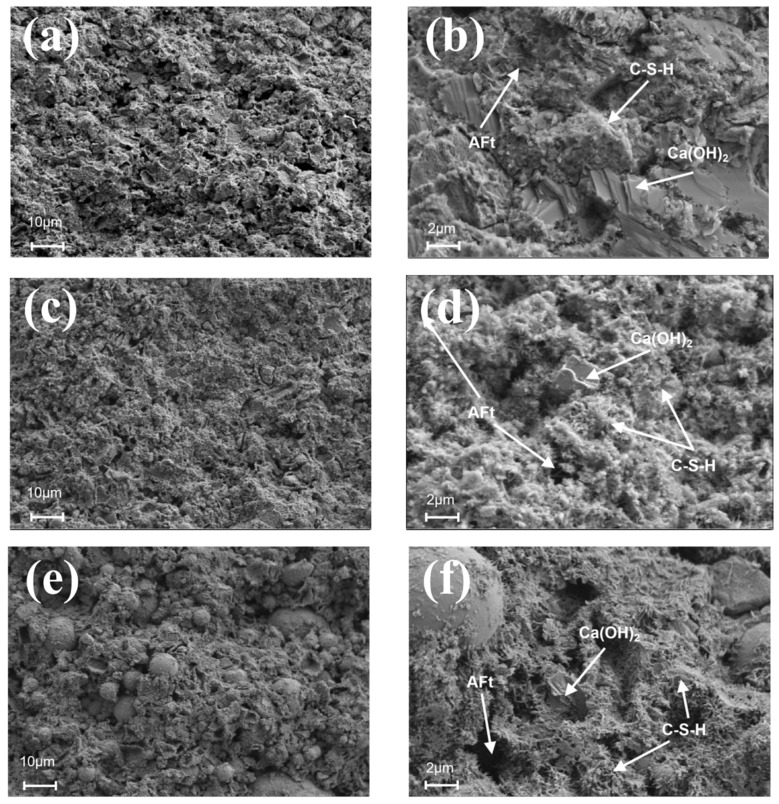
Cement at different UFA dosages at a curing age of 28 d. (**a**) Pure cement (1000×), (**b**) pure cement (5000×), (**c**) cement with 25% UFA (1000×), (**d**) cement with 25% UFA (5000×), (**e**) cement with 50% UFA (1000×), and (**f**) cement with 50% UFA (5000×).

**Figure 11:**
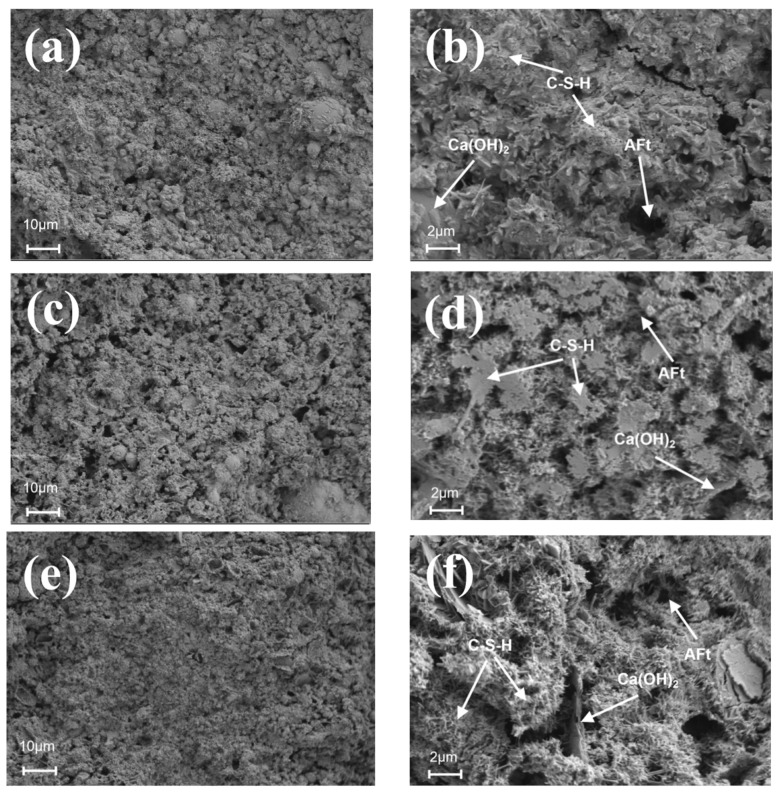
Cement with different NS dosages at 28 d maintenance age. (**a**) Cement with 0.5% NS (1000×), (**b**) cement with 0.5% NS (5000×), (**c**) cement with 1% NS (1000×), (**d**) cement with 1% NS (5000×), (**e**) cement with 1.5% NS (1000×), and (**f**) cement with 1.5% NS (5000×).

Figure 10 and Figure 11 depict the SEM images of different dosages of UFA and NS cement hydrated for 28 d. Figure 10a,b illustrate that the surface of the pure cement paste is loose and porous with many plate-like CH crystals in its interior, surrounded by C-S-H, affecting considerably the paste’s strength. In contrast, Figure 10c–f shows the cement pastes doped with 25% and 50% UFA. As shown in Figure 10e, there is a large number of fly ash balls on the surface of cement with a dosage of 50% UFA. These fly ash balls have a spherical shape and are evenly filled in the voids of the cement slurry, which reduces the porosity of the slurry and improves its density. Additionally, numerous needle-like ettringite (AFt) formations are observed in the voids between the C-S-H gels, but this is not conducive to the development of cement strength. Previous studies have also found that there are a significant number of unhydrated FA particles, as well as pores and cracks, in FA mortar, which have a certain negative impact on its mechanical properties [63].

As show in Figure 11a,c,e, the surfaces of cement paste containing NS exhibit smaller pore structures and higher densifications than those of cement paste without NS. This is because NS not only fills the pores effectively between the cement particles but also promotes the hydration reaction of the cement owing to its high surface activity, resulting in a denser and more homogeneous cement matrix. However, as shown in Figure 11b,d,f, the hydration products gradually increase as a function of the increase in the NS content, and the number of AFt and CH crystals decreases, and only a small number of CH crystals exist in the pores between the C-S-H gels. This is because NS reacts with the CH crystals in a volcanic ash reaction to produce many block-shaped C-S-H gels, indicating that a moderate NS amount can limit the development of CH crystals [64]. Previous studies also demonstrated that NS can promote the formation of C-S-H in cement-based composite materials [60]. These micrographs indicate that the C-S-H gels are stacked on top of each other with interlocking growth, and most of the pores are filled with hydration products to form a denser structure, which enhances strength and densification. In previous studies, SEM experiments revealed similar findings; compared to cement paste containing 0.5% NS, the paste with 1.5% NS showed a marked decrease in spherical FA particles and exhibited higher strength [65].

### 3.5. X-Ray Diffraction Test

**Figure 12:**
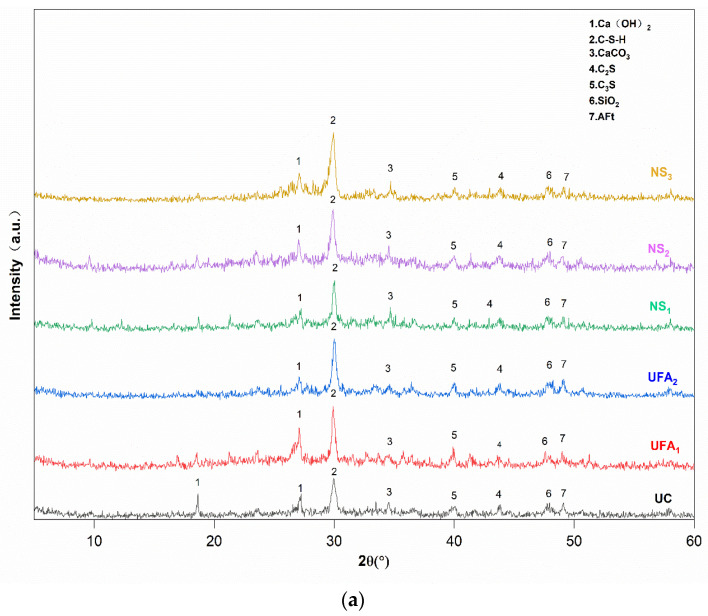

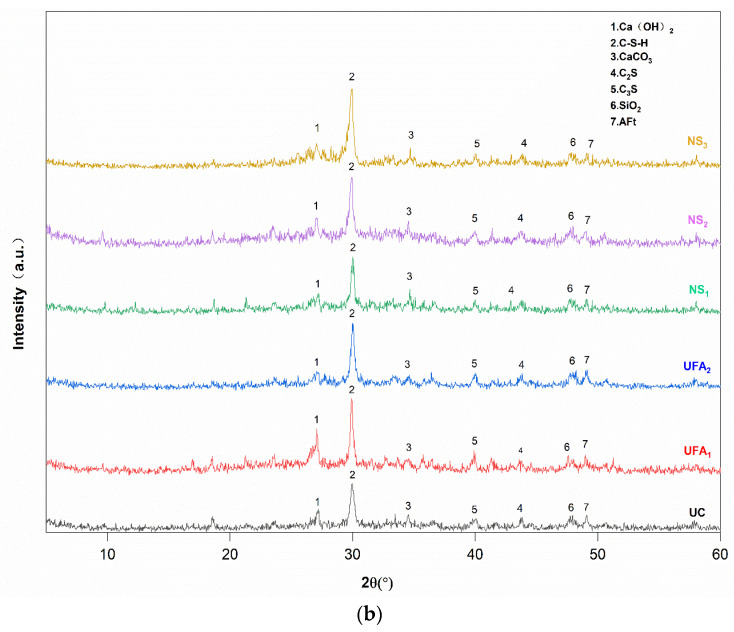
X-ray diffraction (XRD) patterns of hydrated cement at (**a**) 7 d and (**b**) 28 d.

The XRD patterns after 7 d and 28 d of maintenance are shown in Figure 12a,b. The XRD patterns reveal some of the expected hydration products, including CH, CaCO_3_, AFt, C-S-H, and calcium silicate phases (C_2_S and C_3_S). Regarding the UC group, the mapping after 7 d of hydration shows a high intensity of the main peak of AFt, with comparable peak intensities for CH and CaCO_3_; the latter peaks were produced via the partial carbonation of CH. As indicated by the 28 d XRD mapping, CH decreased following an increase in the curing time.

Figure 12 shows that for the slurry containing only UFA, a considerable amount of CaCO_3_ and CH is observed after 7 d of hydration, and the content of CH increases as a function of the increase in UFA dosage. The peak strength of CH after 28 d of hydration is notably lower, while the peak of C-S-H is considerably higher. This is attributed to the volcanic ash reaction between FA and CH, generating C-S-H in the later stages of hydration, thereby consuming some of the CH. Compared with other experimental groups, the CH peak decreased at 7 d and 28 days after the addition of NS, and the C-S-H peak increased. This is the same as the conclusion obtained from previous research [66]. This is because NS and CH continuously undergo pozzolanic reactions to form C-S-H gel again while the cement hydrates to form C-S-H gel. Furthermore, because UFA is mixed in the slurry, CH generated in the early stage of hydration provides a good alkaline environment for UFA, which promotes the pozzolanic reaction between UFA and cement in the late stage of hydration to generate C-S-H gel [67]. Previous research findings indicate [68] that NS can react with CH, increasing the content of C-S-H gel, thereby improving the morphology, uniformity, and density of the C-S-H gel. And, NS can transform the morphology of CH crystals from loose, porous plate-like structures to stable, columnar forms. Moreover, it can be observed that the peak value of C-S-H increases at increased NS doping levels.

### 3.6. Orthogonal Test Analysis

**Figure 13:**
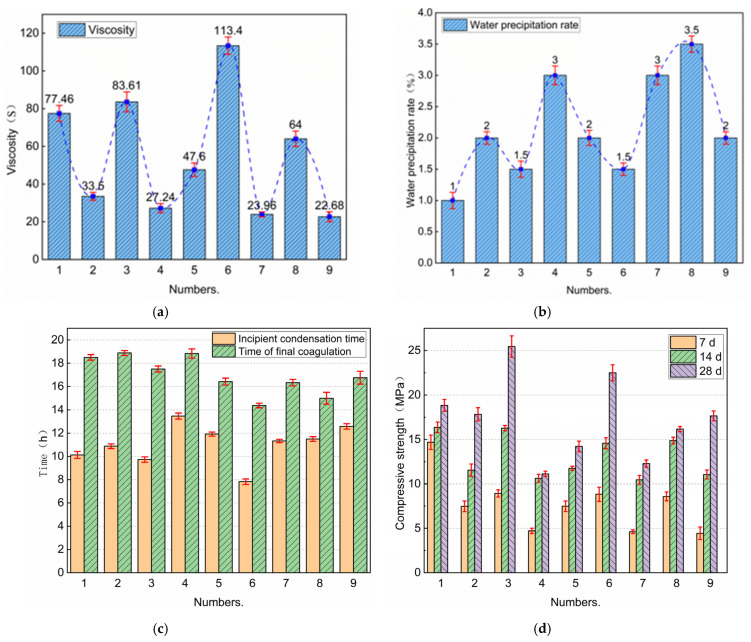
Orthogonal test results: (**a**) viscosity, (**b**) water separation rate, (**c**) setting time, and (**d**) compressive strength.

The analysis suggests that incorporating 50% UFA negatively affects cement performance. Consequently, an orthogonal experimental design was proposed with the UFA and NS contents capped at 50% and 1.5%, respectively. This experiment utilized three factors at three levels, employing the orthogonal experimental design plan “L9 (3^3^)”, as shown in Table 4. To determine the optimal proportion of NS-modified composite cement slurry to achieve the best grouting effect, the evaluation indices set in this test were the viscosity, water precipitation rate, setting time, and compressive strength of the slurry. Viscosity, water precipitation rate, condensation time, and compressive strength tests were conducted following the method described in “Section 2.2”. Table 5 lists the orthogonal test results. Comparisons of the viscosity, water precipitation rate, setting time, and compressive strength for each group of tests are shown in Figure 13a–d.

**Figure 14:**
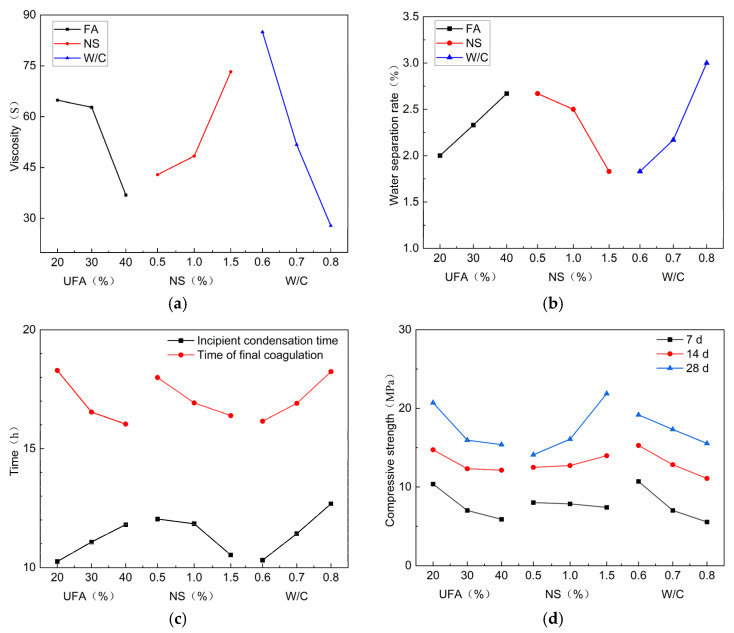
Effects of UFA, NS, and W/C on the slurry performances: (**a**) viscosity, (**b**) water precipitation rate, (**c**) setting time, and (**d**) compressive strength.

An analysis of extreme differences was conducted for each group of test results. The analyzed results are listed in Table 6 and Table 7. Figure 14 shows the trends of the influences of the UFA, NS, and W/C content on the viscosity, water separation rate, setting time, and compressive strength of the slurry. The order of influence on the viscosity and water precipitation rate is W/C > NS > UFA, the order of influence on the initial setting time is W/C > NS > UFA, the order of the final setting time is UFA > W/C > NS, the order of influence on the strength of 7 and 14 d is W/C > UFA > NS, and the order of influence on the 28 d strength is NS > UFA > W/C. 

Considering that this is a multi-index orthogonal experiment, a comprehensive scoring method was utilized to determine the optimal ratio for the NS-modified composite cement slurry. The experiment did not consider the interaction between various factors. The results (detailed in Table 8) indicate that the overall performance of the slurry is mostly influenced by NS, followed by UFA and W/C. Consequently, the optimal slurry compositions were found to be 20% UFA, 1.5% NS, and a W/C ratio of 0.6.

### 3.7. Engineering Applications

#### 3.7.1. Project Overview

**Figure 15:**
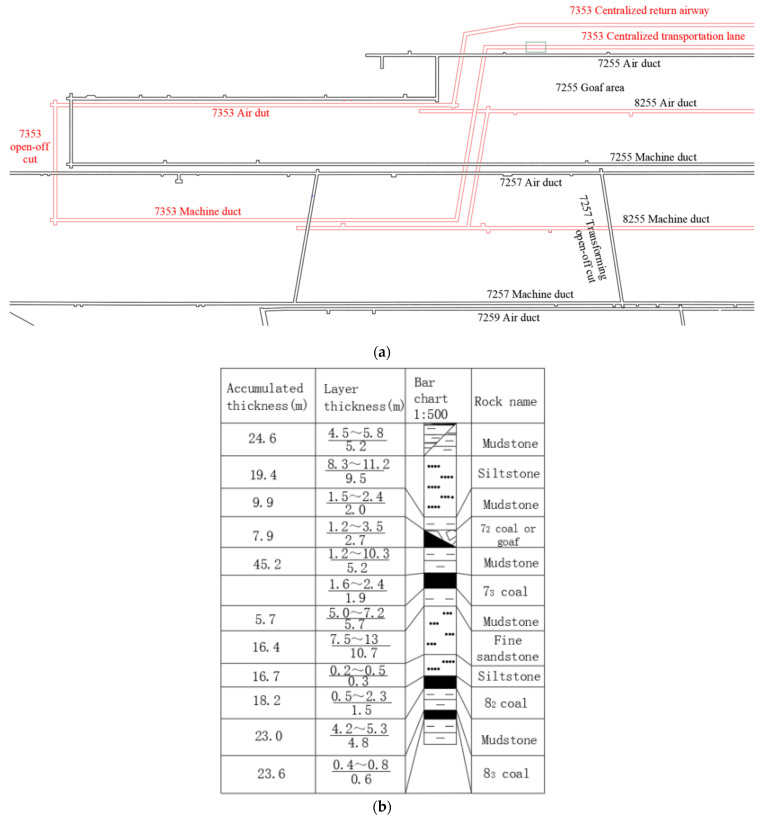
Project overview: (**a**) “7_3_53” working face plan and (**b**) rock comprehensive column chart.

Renlou Coal Mine is situated in Suzhou City in the Province of Anhui and is part of the first section of the southern wing of the middle-fifth mining area. The layout of the working face follows a “single-side, two-lane” configuration, with the air and machine ducts ranging in elevation from −340 to −407.5 m and from −388 to −446 m, respectively, to facilitate U-shaped ventilation. The primary coal seam at the working face is the “7_3_” coal seam, which is directly overlain by mudstone with an average thickness of 5.2 m, succeeded upward by either the “7_2_” coal seam or goaf. The floor of the working face comprises mudstone with an average thickness of 5.7 m. The centralized return airway of the “7_3_53” working face is located near the F5 reverse fault protection coal pillar line, indicating a complex geological structure. Figure 15a,b presents the plan of working face “7_3_53” along with a comprehensive stratigraphic column chart.

#### 3.7.2. Grouting Scheme

In Figure 15a, the green marked locations are the grouting sites. During an onsite investigation, it was observed that the fault compromised considerably the integrity of the tunnel roof. To reinforce the structure, both shallow and deep-hole grouting techniques were employed. The section marked in green on the diagrams served as the experimental area. For this segment, five grouting pipes were installed in the arch section, four in the support section, and three in the bottom plate, totaling 12 grouting pipes across the section.

The drilling involves alternating shallow and deep holes with a spacing of 1000 mm between them. The details of the grouting parameters are as follows:

(1) Shallow hole grouting adopts hollow grouting pipes with the specifications of Φ20 mm × 2000 mm. The depth of each hole is 2000 mm, with an allowable leakage length of 200 mm. The grouting pipes are spaced 2000 mm apart, arranged in rows with a grouting pressure ranging from 1 to 3 MPa. The grouting hole at the top of the tunnel is centrally located in the curved roof with additional holes symmetrically placed on both sides.
(2) The grouting depth for the top deep hole is 6000 mm. Two connected hollow grouting pipes are used with dimensions of Φ20 mm × 3000 mm each. Similarly to shallow grouting, the allowable leakage length is 200 mm, with the same spacing of 2000 mm × 2000 mm between the successive rows. The grouting pressure for these deep holes is set between 4 and 6 MPa. The principal deep hole is centrally positioned in the tunnel’s curved roof, with others symmetrically arranged at 2000 mm intervals. The bottom plate grouting also employs hollow pipes with an overall drilling depth of 5000 mm, combining pipes of Φ20 mm × 3000 mm and Φ20 mm × 2000 mm dimensions, maintaining the same leakage and spacing standards.
(3) The grouting uses ultrafine cement graded P · O42.5, mixed at a slurry ratio of UFA 20%, NS 1.5%, and a W/C of 0.6.

#### 3.7.3. Grouting Effect

**Figure 16:**
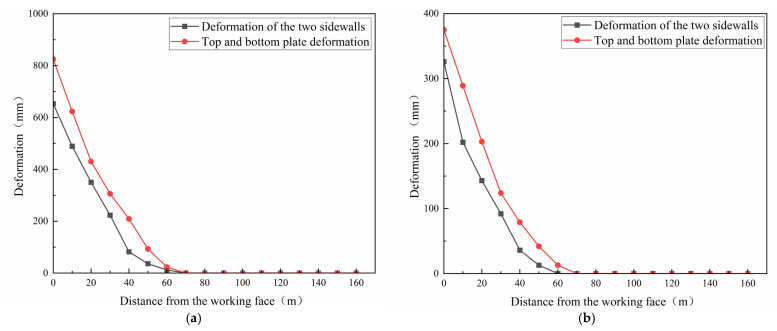
Deformations of the two sidewalls and deformations of the top and bottom plates: (**a**) before grouting and (**b**) after grouting.

To evaluate the effectiveness of the grouting, the displacement of the surrounding rock surface on the roof, floor, and both sides of the tunnel was measured before and after the grouting process. As shown in Figure 16, before grouting, the deformation predominantly occurred within 60 m of the working face, where the surrounding rock exhibited severe deformation. The maximum deformation recorded on the sides was 653 mm, and on the top and bottom plates, it reached 825 mm. Following the grouting process, there was a notable reduction in deformation amplitude; the maximum deformation on the sides decreased to 326 mm, and on the top and bottom plates it reduced to 375 mm. These measurements confirm that the grouting effectively filled the voids within the fractured rock masses, markedly improved the stability of the surrounding rock, and underscored the success of grouting as a reinforcement measure to control tunnel deformation.

## 4. Conclusions

In this study, the effects of NS on the physical properties and microstructural development of UC grouting materials with low UFA dosages were investigated. The optimum proportions for the modified cement composite slurry in this test range were determined using orthogonal and industrial tests. The following conclusions can be drawn from the results of this study:

1. The inclusion of 25% UFA in the cement slurry progressively reduced viscosity and enhanced fluidity. At a UFA content of 50%, the viscosity decreased by 91%, the water precipitation rate increased by 3.77%, and the compressive strength decreased by 51% and 29.2% at 7 and 28 d, respectively, indicating detrimental effects on the performance of grout injections.
2. The addition of NS to the UC and UFA composites compensated for the lower strength in the early stage, thus ensuring the enhancement of the slurry performance. The optimal results were observed when the NS content was at 1.5%, leading to a decrease in the water precipitation rate by 0.9%, an increase in the viscosity by 135.2%, and an enhancement in the compressive strength by 51.2% and 37% at 7 and 28 d, respectively.
3. The microscopic experiments revealed that the addition of UFA and NS reduced the porosity of the slurry surface. As the NS content increased, the intensity of the diffraction peak of the C-S-H gel also increased and peaked at an NS content of 1.5%. However, the presence of a large number of fly ash particles on the surface of the slurry with 50% UFA negatively impacted the slurry’s performance.
4. Orthogonal testing determined that the order of influence on slurry performance in the UFA and NS composite was W/C > UFA > NS. Utilizing a comprehensive scoring method, the optimal slurry composition was established as UFA 20% and NS 1.5%, with a W/C of 0.6. This composition ensures that the slurry possesses robust overall performance, effective penetration into microcracks, and the ability to handle diverse engineering environments.
5. Industrial tests conducted at the “7_3_53” working face of Renlou Coal Mine demonstrated that the on-site grouting effectively reinforced the tunnel structures. After grouting, the maximum deformation of the top and bottom plates and the sides was reduced to 375 mm and 326 mm, respectively. After the grouting, no occurrence of secondary deformation was observed at the “7_3_53” working face, indicating that the slurry was highly effective in managing tunnel deformation and reinforcing the structures.

While this study focused on the effects of low UFA and NS contents on the physical properties and microstructural development of ultrafine cement grouting materials, future research should explore the implications of using higher dosages of UFA and NS. These studies will help elucidate the effects on the physical properties of the cement slurry and the structural characteristics of the hydration products across a range of dosages.

## Figures and Tables

**Table 1:** Chemical compositions of nanosilica, ultrafine cement, and ultrafine fly ash.

Type	CaO	SiO_2_	Al_2_O_3_	Fe_2_O_3_	Mg_2_O	Na_2_O	K_2_O	TiO_2_	LOSS
UC	7.6%	48%	27%	3.5%	4.2%	−	5.6%	−	4.1%
UFA	55.1%	22.1%	7.1%	6.4%	−	3.5%	2.3%	−	3.5%
NS	−	99.9%	0.0031%	0.002%	−	−	−	0.002%	−

**Table 2:** Mix proportion design of NS and UFA slurries at different contents.

No.	UC Content (%)	UFA Content (%)	NS Content (%)	W/C	Water-Reducing Agent Content (%)
1	99	0	0.0	0.6	1
2	74.5	24.5	0.0	0.6	1
3	49.5	49.5	0.0	0.6	1
4	74.25	24.25	0.5	0.6	1
5	74	24	1.0	0.6	1
6	73.75	23.75	1.5	0.6	1

**Table 3:** Physical performance test results of NS and UFA slurries at different contents.

No.	Water Separation Rate (%)	Viscosity (s)	Compressive Strength (MPa)		
			7 d	14 d	28 d
1	1.23	303	11.82	16.82	18.23
2	2	54	6.94	11.24	14.37
3	5	27.19	5.79	10.55	12.91
4	1.8	72.57	7.68	12.12	15.47
5	1.5	98.46	8.03	13.98	16.89
6	1.1	170.65	10.49	15.51	19.68

**Table 4:** Orthogonal experimental design table.

No.	UFA Content (%)	NS Content (%)	W/C	Water-Reducing Agent Content (%)
1	20%	0.5%	0.6	1%
2	20%	1%	0.8	1%
3	20%	1.5%	0.7	1%
4	30%	0.5%	0.8	1%
5	30%	1%	0.7	1%
6	30%	1.5%	0.6	1%
7	40%	0.5%	0.7	1%
8	40%	1%	0.6	1%
9	40%	1.5%	0.8	1%

**Table 5:** Orthogonal test results.

No.	Incipient Condensation Time (h)	Time of Final Coagulation (h)	Water Separation Rate (%)	Viscosity (s)	Compressive Strength (MPa)		
					7 d	14 d	27 d
1	10.13	18.50	1	77.46	14.69	16.37	18.84
2	10.88	18.88	2	33.5	7.47	11.54	17.84
3	9.73	17.50	1.5	83.61	8.93	16.28	25.45
4	13.47	18.83	3	27.24	4.71	10.63	11.13
5	11.92	16.42	2	47.6	7.48	11.75	14.22
6	7.83	14.38	1.5	113.4	8.83	14.58	22.49
7	11.33	16.33	3	23.96	4.62	10.46	12.29
8	11.5	15.00	3.5	64	8.59	14.88	16.18
9	12.58	16.75	2	22.68	4.44	11.06	17.66

**Table 6:** Analysis of extreme differences in slurry performance.

Characteristic	Variable	k1	k2	k3	R	Priority of Factors
Viscosity	UFA	64.86	62.75	36.88	27.98	W/C > NS > UFA
	NS	42.89	48.37	73.23	30.34	
	W/C	84.95	51.72	27.81	57.15	
Separation rate test	UFA	2.00	2.33	2.67	0.67	W/C > NS > UFA
	NS	2.67	2.50	1.83	0.83	
	W/C	1.83	2.17	3.00	1.17	
Incipient condensation time	UFA	10.25	11.07	11.80	1.56	W/C > NS > UFA
	NS	11.64	11.43	10.05	1.60	
	W/C	9.82	10.99	12.31	2.49	
Time of final coagulation	UFA	18.29	16.54	16.03	2.27	UFA > W/C > NS
	NS	17.89	16.77	16.21	1.68	
	W/C	15.96	16.75	18.15	2.19	

**Table 7:** Analysis of extreme differences in the compressive strengths of the slurries.

Characteristic	Variable	k1	k2	k3	R	Priority of Factors
Compressive strength at 7 d	UFA	10.36	7.01	5.88	4.48	W/C > UFA > NS
	NS	8.01	7.85	7.40	0.61	
	W/C	10.70	7.01	5.54	5.16	
Compressive strength at 14 d	UFA	14.73	12.32	12.13	2.60	W/C > UFA > NS
	NS	12.49	12.72	13.97	1.49	
	W/C	15.28	12.83	11.08	4.20	
Compressive strength at 28 d	UFA	20.71	15.95	15.38	5.33	NS > UFA > W/S
	NS	14.09	16.08	21.87	7.78	
	W/C	19.17	17.32	15.54	3.63	

**Table 8:** Results of the extreme variance analysis of the composite scoring method.

Number	UFA	NS	W/C	D	Comprehensive Score
1	1	1	1	1	206.89
2	1	2	3	2	138.96
3	1	3	2	3	213.66
4	2	1	3	3	115.47
5	2	2	2	1	144.84
6	2	3	1	2	228.91
7	3	1	2	2	109.36
8	3	2	1	3	173.3
9	3	3	3	1	120.33
K1	559.51	431.72	609.1	472.06	
K2	489.22	457.1	467.86	477.23	
K3	402.99	562.9	374.76	502.43	
k1	186.50	143.91	203.03	157.35	
k2	163.07	152.37	155.95	159.08	
k3	134.33	187.63	124.92	167.48	
R	52.17	43.73	78.11	10.12	
Priority of factors	W/C > UFA > NS				

## Data Availability

Data is contained within the article.
